# Comparison of the Uptake of Hepatocellular Carcinoma on Pre-Therapeutic MDCT, CACT, and SPECT/CT, and the Correlation with Post-Therapeutic PET/CT in Patients Undergoing Selective Internal Radiation Therapy

**DOI:** 10.3390/jcm10173837

**Published:** 2021-08-26

**Authors:** Timo C. Meine, Thomas Brunkhorst, Thomas Werncke, Christian Schütze, Arndt Vogel, Martha M. Kirstein, Cornelia L. A. Dewald, Lena S. Becker, Sabine K. Maschke, Nils Kretschmann, Frank K. Wacker, Jan B. Hinrichs, Bernhard C. Meyer

**Affiliations:** 1 Institute for Diagnostic and Interventional Radiology, Hannover Medical School, Carl-Neuberg-Straße 1, 30625 Hannover, Germany; meine.timo@mh-hannover.de (T.C.M.); werncke.thomas@mh-hannover.de (T.W.); dewald.cornelia@mh-hannover.de (C.L.A.D.); becker.lena@mh-hannover.de (L.S.B.); maschke.sabine@mh-hannover.de (S.K.M.); nils.kretschman@stud.mh-hannover.de (N.K.); wacker.frank@mh-hannover.de (F.K.W.); meyer.bernhard@mh-hannover.de (B.C.M.); 2 Department of Nuclear Medicine, Hannover Medical School, Carl-Neuberg-Straße 1, 30625 Hannover, Germany; brunkhorst.thomas@mh-hannover.de; 3 Department of Nuclear Medicine, University Medical Center Goettingen, Robert-Koch-Straße 40, 37075 Goettingen, Germany; christian.schütze@med.uni-goettingen.de; 4 Department of Radiation Protection and Medical Physics, Hannover Medical School, Carl-Neuberg-Straße 1, 30625 Hannover, Germany; 5 Department of Hepatology, Gastroenterology and Endocrinology, Hannover Medical School, Carl-Neuberg-Straße 1, 30625 Hannover, Germany; vogel.arndt@mh-hannover.de; 6 1st Department of Medicine, University Medical Center Schleswig-Holstein Luebeck Campus, Ratzeburger Allee 160, 23562 Luebeck, Germany; martha.kirstein@uksh.de

**Keywords:** liver, hepatocellular carcinoma (HCC), selective internal radiation therapy (SIRT), multi-detector computed tomography (MDCT), C-arm computed tomography (CACT), single-photon emission computed tomography/computed tomography (SPECT/CT), positron emission tomography/computed tomography (PET/CT), Yttrium^90^ (Y90)

## Abstract

(1) Background: To comparatively analyze the uptake of hepatocellular carcinoma (HCC) on pre-therapeutic imaging modalities, the arterial phase multi-detector computed tomography (MDCT), the parenchymal phase C-arm computed tomography (CACT), the Technetium^99^m-macroaggregates of human serum albumin single-photon emission computed tomography/computed tomography (SPECT/CT), and the correlation to the post-therapeutic Yttrium^90^ positron emission tomography/computed tomography (PET/CT) in patients with selective internal radiation therapy (SIRT). (2) Methods: Between September 2013 and December 2016, 104 SIRT procedures were performed at our institution in 74 patients with HCC not suitable for curative surgery or ablation. Twenty-two patients underwent an identical sequence of pre-therapeutic MDCT, CACT, SPECT/CT, and post-therapeutic PET/CT with a standardized diagnostic and therapeutic protocol. In these 22 patients, 25 SIRT procedures were evaluated. The uptake of the HCC was assessed using tumor-background ratio (TBR). Therefore, regions of interest were placed on the tumor and the adjacent liver tissue on MDCT (TBR^MDCT^), CACT (TBR^CACT^), SPECT/CT (TBR^SPECT/CT^), and PET/CT (TBR^PET/CT^). Comparisons were made with the Friedman test and the Nemenyi post-hoc test. Correlations were analyzed using Spearman’s Rho and the Benjamini–Hochberg method. The level of significance was *p* < 0.05. (3) Results: TBR on MDCT (1.4 ± 0.3) was significantly smaller than on CACT (1.9 ± 0.6) and both were significantly smaller compared to SPECT/CT (4.6 ± 2.0) (p*Friedman-Test* < 0.001; p*TBR^MDCT^/TBR^CACT^* = 0.012, p*TBR^MDCT^/TBR^SPECT/CT^* < 0.001, p*TBR^CACT^/TBR^SPECT/CT^* < 0.001). There was no significant correlation of TBR on MDCT with PET/CT (r*TBR^MDCT^/TBR^PET/CT^* = 0.116; *p* = 0.534). In contrast, TBR on CACT correlated to TBR on SPECT/CT (r*TBR^CACT^/TBR^SPECT/CT^* = 0.489; *p* = 0.004) and tended to correlate to TBR on PET/CT (r*TBR^CACT^/TBR^PET/CT^* =0.365; *p* = 0.043). TBR on SPECT/CT correlated to TBR on PET/CT (r*TBR^SPECT/CT^/TBR^PET/CT^* = 0.706; *p* < 0.001) (4) Conclusion: The uptake assessment on CACT was in agreement with SPECT/CT and might be consistent with PET/CT. In contrast, MDCT was not comparable to CACT and SPECT/CT, and had no correlation with PET/CT due to the different application techniques. This emphasizes the value of the CACT, which has the potential to improve the dosimetric assessment of the tumor and liver uptake for SIRT.

## 1. Introduction

Selective internal radiation therapy (SIRT) with Yttrium^90^ (Y90) microsphere is a transarterial liver-directed therapy used to treat primary and secondary hepatic malignancies [1,2]. Current studies confirmed the safety of SIRT, but the efficacy was not superior to conventional medical treatment in patients with advanced hepatocellular carcinoma [3,4,5]. It was shown that the tumor dose is most essential for the response rate and overall survival in patients undergoing SIRT [6]. Hence, partition models were developed to improve the dose calculation, which showed for glass microspheres that a tumoral dose threshold of >205 Gy was required for sufficient tumor response and normal liver should not be exposed to >60–120 Gy [6,7]. Thus, accurate calculation of the dose distribution is most important for an effective SIRT procedure.

Calculation of the dose distribution is classically performed in a pre-therapeutic evaluation session supra-selectively administering Technetium^99^m-macroaggregates of albumin (Tc^99^m-MAA) or -human serum albumin (Tc^99^m-HSA) in the liver artery of interest followed by single-photon emission computed tomography/computed tomography (SPECT/CT) [8]. The uptake of Tc^99^m-MAA or -HSA in the tumor in relation to non-tumoral liver tissue represents the relative hypervascularization and is the key for the dose calculation in partition models [7]. Partition models depend on both volumes and activities of the tumor and the target liver tissue [7]. The gold standard for volume assessment involves the segmentation of tumor and target liver tissue on pre-therapeutic magnetic resonance imaging or multi-detector computed tomography, which has recently shown to be comparable to segmentations on C-arm computed tomographies [9,10,11]. Tumor and liver iodine uptake on arterial phase computed tomography correlated with Tc^99^m-MAA uptake in SPECT/CT [12]. In addition, a territory model based on arterial phase multi-detector computed tomography (MDCT) was promising for the simulation of the dose distribution [13]. Relative contrast differences are not only detectable using MDCT but also using C-arm computed tomography acquired in the parenchymal phase (CACT) [14,15]. The intra-arterial injection of contrast medium to generate the CACT enables the detection of hypervascularized tumors in the exclusively arterial phase without portal vein contrast enhancement. Therefore, we hypothesize that the iodine uptake of the tumor and the non-tumoral liver tissue on CACT in patients undergoing SIRT corresponds to the activities measured with SPECT/CT and PET/CT and is superior to MDCT. If true, this could improve the interventional workflow, and the detection and uptake assessment of small tumors, since CACT has a higher spatial resolution compared to MDCT and SPECT/CT.

## 2. Materials and Methods

### 2.1. Study Population

Between September 2013 and December 2016, 104 SIRT treatment sessions in 74 patients were retrospectively reviewed at or Picture Archiving Computational System and Radiology Information System for study inclusion. Inclusion criteria were patients > 18 years of age with HCC not eligible for curative surgery or local ablative therapies, according to the Barcelona Clinic Liver Cancer (BCLC) staging system and a positive vote for SIRT in our interdisciplinary tumor board [2]. Further, technical inclusion criteria were an identical scanner sequence with dedicated image acquisition parameters of the SIRT procedures with pre-therapeutic MDCT, CACT, and SPECT/CT, and post-therapeutic PET/CT. Exclusion criteria were different/external imaging and substantial tumor change between the acquisition of the MDCT and the CACT, according to lesion-based modified Response Evaluation Criteria in Solid Tumors (mRECIST) [16]. Overall, 22 patients (17 men and 5 women between 71 ± 9 years) and 25 SIRT treatment sessions with 31 HCC manifestations with a viable tumor diameter of 4.8 ± 2.5 cm were included. A single SIRT procedure had to be excluded because of lesion-based progression, according to mRECIST, with an increase in viable tumor diameter of +119% between MDCT and CACT. Time interval between MDCT and CACT was 40 ± 37 days and between pre-therapeutic SPECT/CT and post-therapeutic PET/CT it was 16 ± 4 days. Demographic data were recorded.

### 2.2. Image Acquisition

#### 2.2.1. MDCT

All patients underwent pre-therapeutic multi-detector computed tomography with an arterial phase of the liver and a portal venous phase of the abdomen using the same scanner (GE LightSpeed VCT, GE Healthcare, Chicago, IL, USA). Images were acquired in breath-hold after intravenous administration of 1.5 mL/kg iodinated contrast material (Iohexol 350 mg I/L; GE Healthcare, Vélizy-Villacoublay, France) at a rate of 2.5 mL/s using an automatic injector. The arterial phase was generated with an automated trigger in the aorta and the portal phase was acquired at 80 s after the beginning of contrast material administration. Images were obtained with the following acquisition parameters: 120 kV; Care Dose^®^ System (GE Healthcare, Chicago, IL, USA) mA; beam pitch configuration, 32 × 0.625 mm; rotation speed, 0.5 s; pitch, 0.8; matrix of 512 × 512, slice thickness, 1 mm; and reconstruction interval, 0.7 mm.

#### 2.2.2. CACT

During pre-therapeutic SIRT evaluation sessions, all patients were examined with an arterial and a parenchymal phase C-arm computed tomography performed by experienced interventional radiologists (JBH, BCM) on the same monoplane, ceiling-mounted angiographic system (Artis Q, Siemens Healthcare, Forchheim, Germany). At the day of the SIRT evaluation, the patient received 4 mL of sodium perchlorate before the intervention to block gastric uptake of ^99^mTc-HSA. Under local anesthesia and ultrasound guidance, the right common femoral artery was assessed and a mesentericoportography was obtained. Afterwards, a sequential acquisition of a hepatic angiography and a C-arm computed tomography was acquired to analyze tumor-feeding arteries and to plan an adequate supra-selective catheter position in a breath hold of 10 s. If necessary, coil embolization of extrahepatic arteries was performed. Finally, a microcatheter (Maestro^®^ Microcatheter, Merit Medical Systems, South Jordan, UT, USA) was placed in a supra-selective position in the appropriate hepatic artery. A C-arm computed tomography was acquired in a parenchymal phase with injection of 50 mL of contrast mixture (150 mg I/mL; 50% Iomeperol 300 mg I/L Bracco Imaging, Milan, Italy; and 50% NaCl 0.9%, B. Braun, Melsungen, Germany) at a flow rate of 2.5 mL/s and with an injection time of 20 s. Afterwards, a mean dose of 148 mega Becquerel (MBq) (±8) of ^99^mTc-HSA, followed by 20 mL of saline (NaCl 0.9%, B. Braun, Melsungen, Germany), were manually injected through the microcatheter by a nuclear medicine specialist (T.B.). C-arm computed tomography images were generated with the following acquisition parameters: 100 kV, 6-s acquisition time; 60 projections/s, 396 projections, matrix of 480 × 616, and a slice thickness of 0.49 mm. Complication of pre-therapeutic SIRT evaluation sessions were recorded.

#### 2.2.3. SPECT/CT

The pre-therapeutic SPECT/CT was acquired in the clinic for nuclear imaging within 2 to 3 h after the Tc^99^m-HSA administration on a Siemens Symbia T2 (Siemens Healthineers, Siemens, Erlangen, Germany) with a dual-head gamma camera and an integrated computed tomography scanner. A planar anterior and posterior whole-body scintigraphy were generated and a geometric mean was determined for the calculation of the extrahepatic shunt. Abdominal SPECT/CT imaging was performed on the liver. Single-photon emission computed tomography images were acquired with the following parameters: 120 projections with 10 s per projection over 360 degrees. For reconstruction, we used Iterative reconstruction OSEM with 4 iterations and 8 subsets under a CT-based attenuation correction in a matrix of 128 × 128 with a 4.7-mm slice thickness. Computed tomography acquisition parameters were: 130 kV, 30 mA, 195 slices, 2-mm slice thickness, 2-mm slice distance, matrix of 512 × 512, Field-Of-View 500 × 500 mm^2^, and 5-mm reconstruction thickness. A Tc^99^m-HSA dose for pre-therapeutic SPECT/CT was recorded.

#### 2.2.4. PET/CT

During SIRT treatment sessions, the microcatheter tip was placed in the same supra-selective position as assessed during the pre-therapeutic session. The dose of Y90 microspheres (Theraspheres^®^, BTG, London, UK) was calculated using a partition model identical to SIMPLICIT90Y (https://www.bostonscientific.com/en-EU/products/selective-internal-radiation-therapy/simplicit90y.html (accessed on 30 July 2021)). A dose of 2.34 ± 1.20 Giga-Becquerel (GBq) Y90 microspheres was manually injected through the microcatheter (T.B.). One day after administration of the Y90 microspheres, the dose distribution of the microspheres was assessed by post-therapeutic PET/CT on Siemens Biograph 128 mCT (Siemens Healthineers, Siemens, Erlangen, Germany). Positron emission tomography image acquisition was performed with 2 bedpositions â 10 min with TOF. For reconstruction, we used the TrueX-algorithm (OSEM) with 2 iterations and 21 subsets, and applied correction for attenuation (CT-based), scatter, randoms, and dead-time. Computed tomography images were generated with the following parameters: 120 kV, 100 mA, 117 slices, 5-mm slice thickness, 3-mm slice distance, matrix of 512 × 512, Field-Of-View 780 × 780 mm^2^, and 5-mm reconstruction thickness. Y90 microspheres dose and complications were recorded.

### 2.3. Image Analysis

**Figure 1:**
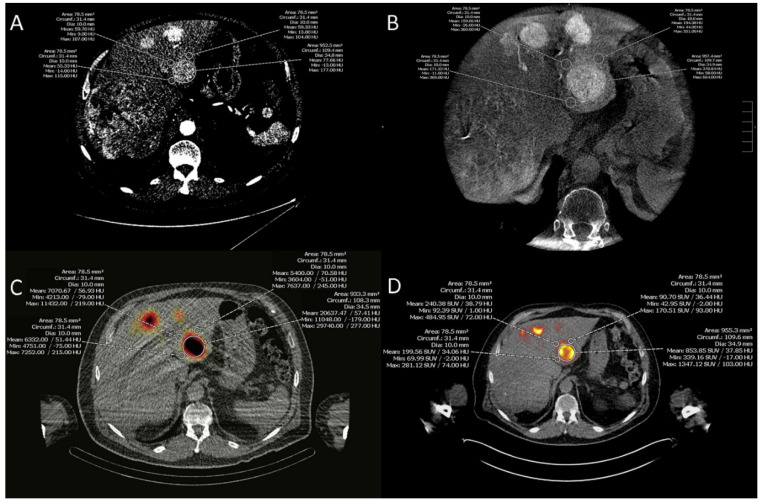
Image analysis. A region of interest was drawn on the tumor and three regions of interest were placed on the adjacent liver tissue in equal positions on arterial phase multi-detector tomography (**A**), parenchymal phase C-arm computed tomography (**B**), Technetium^99^m-macroaggregates of human serum albumin single-photon emission computed tomography and computed tomography (**C**), Yttrium^90^ positron emission tomography and computed tomography (**D**), mean hounsfield units, counts, and becquerel/milliliter were recorded.

Representative tumors detectable with pre-therapeutic MDCT, CACT, SPECT/CT, and post-therapeutic PET/CT were selected for the analysis with minimum diameter of 2 cm and a maximum of three tumors per patient. Images of the described modalities were manually registered in an axial plane focused on the slice position of the tumor using Visage 7.1 (VISAGE Imaging GmbH, Berlin, Germany). A region-of-interest (ROI) was drawn on the tumor and three ROIs were placed on the adjacent liver tissue in equal positions on all modalities (s. Figure 1). Mean hounsfield units, counts and becquerel/milliliter per pixel were recorded. Since noise and resolution properties of radiological and nuclear imaging were different, the tumor-background ratio (TBR) was used for intermodal comparison. The TBR was calculated by dividing the mean tumor uptake value per pixel by the average uptake value of the mean three non-tumoral liver tissue values per pixel, as described by Ihlan et al. [17].

### 2.4. Statistics

Data analysis was conducted with non-parametric statistics, since Gaussian distribution of the data was rejected using the Shapiro–Wilk test. Comparisons of the TBR on pre-therapeutic MDCT, CACT, and SPECT/CT were made with a two-sided Friedman test and conservative Nemenyi post-hoc test. Correlations of the TBR on pre-therapeutic and post-therapeutic imaging modalities were analyzed using a two-sided Spearman Rho correlation, and correction for multiple tests was applied using the Benjamini–Hochberg method due to the exploratory study design. Variables are shown as means ± standard deviation. The level of significance was *p* < 0.05. Statistical analysis was performed using R 3.6.2 statistical computational system with the R package PMCMRplus (https://r-project.org).

## 3. Results 

### 3.1. Study Population

Patients were predominantly male (77%) with a mean age of 70 years. The Child–Pugh-Score was A in 17 cases and B in 5 cases; BCLC stage was B in 11 and C in 11 patients. Portal vein tumor manifestation was present in seven patients. In 16 patients, interventional procedures were performed prior to SIRT, such as transarterial chemoembolization, transarterial chemosaturation, or percutaneous ablation. Details are summarized in Table 1.

### 3.2. Image Acquisition

SIRT procedures were performed as follows: 16 cases unilateral right, six cases unilateral left, two cases bilateral, and one case in the territory of a segment-IV-artery-branch. A dose of 150 ± 9 MBq of ^99^mTc-HSA was administered for SIRT evaluation in the right hepatic lobe and 148 ± 7 MBq of ^99^mTc-HSA for SIRT evaluation in the left hepatic lobe as well as 143 ± 5 MBq for bilobar SIRT evaluation. A total of 1.42 MBq of ^99^mTc-HSA was injected in a segment-IV artery branch once. The calculated and injected doses of Y90 microspheres were 2.69 ± 1.03 GBq for the right, 1.19 ± 0.61 GBq for the left hepatic lobe, 3.45 ± 1.82 GBq for bilobar treatment, and 1.4 GBq in a segment-IV artery branch. All pre-therapeutic imaging modalities—MDCT, CACT, SPECT/CT—and post-therapeutic PET/CT were performed without complications.

### 3.3. Image Analysis

The TBR on MDCT (TBR^MDCT^) was 1.4 ± 0.3, whereas the TBR on CACT (TBR^CACT^) was 1.9 ± 0.6. For the dose planning modality, the SPECT/CT and the TBR (TBR^SPECT/CT^) were 4.6 ± 2.0. On PET/CT, i.e. the gold standard for the dose detection, the TBR (TBR^PET/CT^) was 4.7 ± 4.8. The comparison of the TBR among the pre-therapeutic imaging modalities—MDCT, CACT and SPECT/CT—was statistically significant (p*Friedman-Test* < 0.001). The TBR^MDCT^ and TBR^CACT^ were significantly smaller than the TBR^SPECT/CT^ (p*TBR^MDCT^/TBR^SPECT/CT^* < 0.001 and p*TBR^CACT^/TBR^SPECT/CT^* < 0.001). There was also a significant difference between the TBR on MDCT and CACT (p*TBR^MDCT^/TBR^CACT^* = 0.012). Correlation between TBR^MDCT^ and TBR^SPECT/CT^ or TBR^PET/CT^ were not statistically significant (r*TBR^MDCT^/TBR^SPECT/CT^* = 0.089; *p* = 0.632 and r*TBR^MDCT^/TBR^PET/CT^* = 0.116; *p* = 0.534). In contrast, the correlation of TBR^CACT^ and TBR^SPECT/CT^ was significantly positive (r*TBR^CACT^/TBR^SPECT/CT^* = 0.489; *p* = 0.004). Further TBR^CACT^ correlated to TBR^PET/CT^ (r*TBR^CACT^/TBR^PET/CT^* = 0.365; *p* = 0.043), which was not significant after the Benjamini–Hochberg method. In addition, TBR^CACT^ correlated also to the TBR^MDCT^ (r*TBR^CACT^/TBR^MDCT^* = 0.506; *p* = 0.003). Of course, there was a positive correlation of TBR^SPECT/CT^ and TBR^PET/CT^ (r*TBR^SPECT/CT^/TBR^PET/CT^* = 0.706; *p* < 0.001).

Results are tabulated in Table 2 and Table 3.

## 4. Discussion

Accurate dose calculation is most important for an effective SIRT procedure, which is based on the relative hypervascularization of the tumor compared to the liver [7]. We hypothesized that the iodine uptake of the tumor and the non-tumoral liver tissue on CACT in patients undergoing SIRT corresponds to the activities measured with SPECT/CT and PET/CT and is superior to MDCT due to the intra-arterial application technique. This could improve the interventional workflow, as well as the detection and uptake assessment of the tumor for SIRT.

Reviewing our results, our hypothesis could be confirmed. TBR on CACT was statistically significant higher than TBR on MDCT and showed a statistically significant positive correlation with TBR on SPECT/CT. Of course, there was a statistically significant correlation of the TBR on CACT and the TBR on MDCT, because the image acquisition of a CACT is comparable to the MDCT. Unfortunately, the correlation of the CACT and PET/CT remained not statically significant after the Benjamini–Hochberg method, which could be explained with the limited sample size. Nevertheless, our study demonstrates the agreement of pre-therapeutic CACT with SPECT/CT and potentially post-therapeutic PET/CT in the assessment of tumor and liver uptake. Of course, the correlation of pre-therapeutic SPECT/CT and post-therapeutic PET/CT was strong in our study with a correlation coefficient of 0.7. This is usually reported to be very strong in the literature with correlation coefficients of 0.9 [18], because the activities of the complete tumor and liver volumes were correlated. However, our study results indicate that tumor and liver uptake on CACT might be comparable to the conventional imaging modalities, pre-therapeutic SPECT/CT, and post-therapeutic PET/CT, for calculation and detection of the dose distribution. Interestingly, MDCT was not comparable to CACT in the quantitative assessment of tumor and liver uptake in relation to SPECT/CT and PET/CT.

The discrepancy of the uptake assessment between the MDCT and CACT can be explained by the different iodine contrast application techniques. The iodine contrast medium is injected in a supra-selective position in the hepatic artery for generation of the CACT, whereas it is administered via a superficial brachial vein for the acquisition of the MDCT. Regarding the different catheter diameters, there are different contrast volumes and viscosities and different flow velocities of the contrast medium application; the most important difference is the vascular access. Intra-arterial injection in the hepatic artery leads to contrast enhancement of arterial hypervascularized tissue in the liver, especially HCC, whereas tissue supplied by the portal vein shows no enhancement, as, in contrast to MDCT, no recirculation through the mesenteric vein occurs. An intravenous application of contrast medium leads to arterial contrast enhancement of all intraperitoneal tissues supplied by the aorta, e.g., liver, pancreas, spleen, adrenal glands, kidneys, bladder, and gut. Depending on the blood flow of the specific tissue (e.g., spleen), contrast enhancement of the portal vein can be present on MDCT. This might reduce the TBR obtained from MDCT, because non-tumoral liver tissue is, in contrast, enhanced via the portal vein, e.g., when splenic or gastrointestinal contrast medium transit is fast. The MDCT in our study is acquired at a specific time point with a very low portal venous contrast, as described before by Hinrichs et al. [19]. In contrast, the arterial liver perfusion values in the study by Reiner et al. were obtained from dynamic liver perfusion computed tomography at multiple time points [12]. Therefore, it is conceivable that the TBR derived from a MDCT might not be equivalent to arterial liver perfusion values from dynamic contrast-enhanced MDCT. Thus, the missing correlation of the TBR between MDCT and SPECT/CT and PET/CT might be flow-dependent. Furthermore, the contrast transit of the visceral organs and consecutively of the portal vein is affected by the arterial inflow, the left ventricular ejection fraction, the capillary system, the outflow, and the venous drainage. Especially, patients with liver cirrhosis may have altered capillary resistance and consecutively delayed hepatic perfusion. Since many patients with HCC have an underlying cause for liver cirrhosis, visceral hemodynamics might have an important effect on the assessment of tumor vascularization on MDCT and possibly on CACT and on SPECT/CT.

Overall, tumor vascularization on pre-therapeutic CACT and SPECT/CT were comparable due to the intra-arterial injection technique, whereas MDCT is not comparable due to intravenous contrast application that potentially increases liver enhancement and lowers the TBR. Although tumor and liver volumes can be accurately derived from C-arm computed tomography [9,10,20], the C-arm computed tomography did not improve the dose prediction of the volume-based MIRD calculation model in comparison to SPECT/CT [20]. When state-of-the-art partition models are applied for dose calculation, both volume and uptake of the tumor and liver are required to calculate the appropriate amount of Y90 microspheres to reach the dose threshold for tumor response. In consideration of our results, it is conceivable that both the volume and also the uptake of the tumor and liver can be derived from C-arm computed tomography, which could be facilitated during the SIRT evaluation. Although there a known difficulties of the CACT with a limited field-of-view and catheter or motion artifacts [21], the main advantage of the C-arm computed tomography is a spatial resolution of less than 1 mm, as compared to the camera of the SPECT/CT with 20 mm and the PET/CT with 10 mm. C-arm computed tomography-based dose adaption or calculation would be an improvement for small and multinodular HCC or different tumor entities, e.g., cholangiocarcinoma and metastasis from neuroendocrine tumors or uveal melanoma. In addition, it was recently reported that the dose distribution can be detected with C-arm computed tomography when iodine-labeled Y90 microspheres were used [22]. Hence, it might be possible to perform up-to-date Y90 dosimetry using C-arm computed tomography for calculation and detection of the dose distribution. Of course, SPECT/CT will still be required to calculate the lung-shunt-fraction [23], which is essential for the safety of the partition models. This could be facilitated when a gamma camera is equipped to the C-arm of the angiographic system to generate CACT and SPECT/CT simultaneously during the procedure. However, C-arm computed tomography-based dosimetry has the potential to support the implementation of combined SIRT evaluation and treatment sessions at the same day [24,25], which could improve tumor control and quality of life for the patient in a cost-saving outpatient clinic. Finally, the quantitative assessment of tumor and liver uptake with the CACT could improve the selection and prediction of patients who could benefit from SIRT treatment. This is already a visual impression of the interventionalist in the angio-suite during the SIRT evaluation session. Uptake assessment of CACT could become a quantitative imaging biomarker for survival of the patients undergoing SIRT.

Our study has several limitations. First, the main limitation is the limited number of patients due to the dedicated scanner protocols, which were required for the technical comparison. Second, the difference in particle size, charge, and intravascular behavior between iodine contrast media and Tc^99^m-HSA particles and Y90 microspheres can lead to different tumor and liver uptake, which further limits the agreement. Third, Tc^99^m-HSA particles and Y90 microspheres were manually injected through the diagnostic catheter, which is not comparable to pump injection used for CACT acquisition. Hence, standardization of the image acquisition has to be addressed to improve the initial uptake agreement between CACT and SPECT/CT, and potentially PET/CT.

## 5. Conclusions

In conclusion, our study indicates that the CACT is in agreement with SPECT/CT due to the intra-arterial injection technique, whereas the MDCT with intravenous iodine application has no correlation to the SPECT/CT. Finally, CACT is in potential agreement with PET/CT compared to the correlation of SPECT/CT and PET/CT, whereas MDCT does not correlate with PET/CT. This emphasizes the higher value of the CACT than of the MDCT for the dosimetric assessment of tumor, and liver uptake and selection of the patients, which could benefit from SIRT.

## Figures and Tables

**Table 1:** Study population.

Study Population	
Gender (Male/Female) Age	17/5 70 ± 9
Child-Pugh-Score	
A	17
B	5
BCLC stage	
B	11
C	11
Primary liver disease	
ASH	6
NASH	2
HBV	5
HCV	2
PBC	1
C1-esterase-deficiency	1
Cryptogenic	5
Prior therapy	
Transarterial chemoembolization	42
Transarterial radioembolization	7
Transarterial chemosaturation	1
Microwave/radiofrequency ablation	2

Table 1 shows the demographic data of the study population. Abbreviation: ASH = alcoholic induced steatohepatitis, BCLC = Barcelona Clinic Liver Cancer, NASH = non-alcoholic induced steatohepatitis, HBV = hepatitis B virus, HCV = hepatitis C virus, PBC = primary biliary cirrhosis.

**Table 2:** Comparison of pre-therapeutic imaging modalities.

	Friedman-Test	Nemenyi-Posthoc-Test
TBR^MDCT^/TBR^CACT^		*p* = 0.012
TBR^MDCT^/TBR^SPECT/CT^	*p* < 0.001	*p* < 0.001
TBR^CACT^/TBR^SPECT/CT^		*p* < 0.001

Table 2 shows the results of the comparison of the pre-therapeutic imaging modalities. Abbreviation: MDCT = arterial phase multi-detector tomography, SPECT/CT = Technetium^99^m-macroaggregates of human serum albumin single-photon emission computed tomography and computed tomography, CACT = parenchymal phase C-arm computed tomography, PET/CT = Yttrium^90^ positron emission tomography and computed tomography, TBR = tumor-background ratio.

**Table 3:** Correlation of pre- and post-therapeutic imaging modalities.

	TBR^MDCT^	TBR^CACT^	TBR^SPECT/CT^	TBR^PET/CT^
**TBR^MDCT^**	-	*r* = 0.506 *p* = 0.003 *	r = 0.089 *p* = 0.632	*r* = 0.116 *p* = 0.534
**TBR^CACT^**	*r* = 0.506 *p* = 0.003 *	-	*r* = 0.498 *p* = 0.004 *	*r* = 0.365 *p* = 0.043
**TBR^SPECT/CT^**	*r* = 0.089 *p* = 0.632	*r* = 0.498 *p* = 0.004 *	-	*r* = 0.706 *p* < 0.001 *
**TBR^PET/CT^**	*r* = 0.116 *p* = 0.534	*r* = 0.365 *p* = 0.043	*r* = 0.706 *p* < 0.001 *	-

Table 3 shows the results of the correlation of the pre- and post-therapeutic imaging modalities. Spearman Rho correlation coefficients (*r*) and significance level (*p*) are given. Abbreviation: * = significant results according to the Benjamini–Hochberg method, MDCT = arterial phase multi-detector tomography, SPECT/CT = Technetium^99^m-macroaggregates of human serum albumin single-photon emission computed tomography and computed tomography, CACT = parenchymal phase C-arm computed tomography, PET/CT = Yttrium^90^ positron emission tomography and computed tomography, TBR = tumor-background ratio.

## Data Availability

Data available on request due to privacy/ethical restrictions.
